# Detection of woody breast condition in commercial broiler carcasses using image analysis

**DOI:** 10.1016/j.psj.2020.12.074

**Published:** 2021-01-14

**Authors:** Juan P. Caldas-Cueva, A. Mauromoustakos, X. Sun, Casey M. Owens

**Affiliations:** ∗ Department of Poultry Science, University of Arkansas, Fayetteville 72701, USA; † Agricultural Statistics Laboratory, University of Arkansas, Fayetteville 72701, USA; ‡ School of Biological Science and Food Engineering, Chuzhou University, Anhui, China 239000

**Keywords:** woody breast, processing, carcass grading, image analysis, meat quality

## Abstract

Image analysis could be an objective and rapid method to identify woody breast (**WB**) myopathy and benefit the global poultry industry. The objective of this study was to determine if there are conformational changes that can be used to detect WB characteristics in commercial broiler carcasses across strains, gender, and ages using image analysis. A total of 900 images of male and female broiler carcasses from commercial standard and high breast-yielding strains and 5 ages (6 through 10 wk) were captured before evisceration. These images were processed and analyzed using ImageJ software. Conformational measurements were M0: breast length; M1: breast width in the cranial region; M2: vertical line from the tip of keel to 1/5th of breast length; M3: breast width at the end of M2; M4: angle formed at the tip of keel and extending to outer points of M3; M5: area of the triangle formed by M3 and lines generated by M4; M6: area of the breast above M3; M7: M6 minus M5. Ratios of these measurements were also considered. Intact breast fillets were scored for WB severity based on tactile evaluation. Regardless of strain, sex, and age, M11 (M1/M0), M9 (M3/M2), and M4 had the highest correlation to WB score (*r*_*s*_ ≥ 0.65; *P* < 0.01). Overall, the best validated model (Gen. R^2^ = 0.61) to predict WB included M1, M2, and M3. Using this model, 91% of broiler carcasses were properly classified as normal or WB along with a sensitivity of 71% to detect affected carcasses. Although the predictive performance of models for detecting the WB condition using these measurements was associated with the broiler strain, sex, and age or live weight, these data also support the feasibility of using image analysis to predict WB defect in broiler carcasses. The possible integration of these image measurements into commercial noncontact, nondestructive, and fast in-line vision grading systems would allow processors to identify broilers with WB and potentially sort, provide large-scale information downstream to further processing operations and upstream to live production.

## Introduction

Given the global preference for chicken meat consumption, experts have intensively selected broilers for rapid growth and high yields (Petracci et al., 2019; Hanning et al., 2020); however, these improvements have been associated with increasing and challenging myopathies such as the “wooden” or “woody” breast (**WB**) condition (Mudalal et al., 2015; Petracci et al., 2015). This WB myopathy is one of the main meat quality problems in the global poultry industry that is characterized by a noticeable hardness in the chicken breast fillets (Sihvo et al., 2014; Mudalal et al., 2015; Caldas-Cueva and Owens, 2020). Chicken fillets affected by WB exhibit histological and physicochemical abnormalities that derives in undesired sensory, nutritional, and technological properties (Soglia et al., 2016; Baldi et al., 2019; Petracci et al., 2019). These negative implications coupled with important levels of incidence between 10 and 40% for moderate and severe cases (Hanning et al., 2020) can result in significant economic losses (Kuttappan et al., 2016) and the limited availability of objective, noncontact, and rapid in-line methods to detect this myopathy is a contributing factor (Caldas-Cueva and Owens, 2020).

The identification and classification of broiler breast fillets based on WB severity are typically estimated by subjective muscle palpation evaluation or laborious and time-consuming instrumental laboratory techniques. Thus, some advanced objective and rapid methods have recently been studied for WB detection and sorting such as the computer vision system (Geronimo et al., 2019) and the near-infrared spectroscopy (Wold et al., 2017, 2019; Geronimo et al., 2019) as well as the fusion of optical coherence tomography and hyperspectral imaging (Yoon et al., 2016). However, these methods were developed for deboned breast fillets which do not anticipate potential problems caused by WB myopathy on previous processing operations such as deboning and portioning (Hanning et al., 2020). Thus, the study of potential in-line control technologies for an early detection and objective classification of broiler carcasses by WB severity needs to be investigated. Efforts to meet these needs have been made such as the application of image analysis to predict WB condition in broiler carcasses. Indeed, our previous experiments were focused on the development and evaluation of statistical models for predicting WB condition based on significant relationships between measurements from broiler carcass images and tactile WB scores validated by instrumental compression force values of their corresponding deboned breast fillets (Caldas-Cueva et al., 2021). Nevertheless, further studies are still needed to validate these relationships when broilers from other strains, gender, and ages are included in the experiment because various studies have reported significant differences in terms of occurrence and severity of myopathies such as WB condition between standard and high breast-yielding strains (Petracci et al., 2013; Mazzoni et al., 2015), males and females (Trocino et al., 2015; Brothers et al., 2019) and different ages (Kuttappan et al., 2017; Radaelli et al., 2017) as well as live weights (Cruz et al., 2017). Therefore, the aim of this study was to determine if there are morphometric changes that can be used to detect WB characteristics in commercial broiler carcasses from different strains, gender, and ages using image analysis technique.

## Materials and methods

### Processing of Birds

A total of 900 male (n = 450) and female (n = 450) broiler carcasses from 3 commercial hybrids [named according to their breast yields representing one standard breast-yielding (**SBY**) and 2 high breast-yielding (**HBY**) strains] and 5 ages (6, 7, 8, 9, and 10 wk) were studied. Broilers from ongoing commercial yield trials were processed at the University of Arkansas Poultry Processing Pilot Plant using commercial practices (Mehaffey et al., 2006) with exception that the carcasses were not chilled. The live weight at slaughter and carcass without giblets weight of broilers across strains, gender, and ages are shown in Table 1.

**Table 1:** Processing and meat quality data of broilers: weights, image-based carcass measurements, and woody breast (WB) incidence across strains, gender, and ages.^1^

Age (wk)	Strain^1^	Sex^2^	Live weight (g)	Carcass (WOG)^3^ weight (g)	Fillet weight (g)	WB incidence rate^4^ (%)		Image measurements^5^							
						No	Yes	M0 (cm)	M1 (cm)	M2 (cm)	M3 (cm)	M4 (°)	M5 (cm^2^)	M6 (cm^2^)	M7 (cm^2^)
6	HBY	F	2,371 ± 173	1,813 ± 142	551 ± 62	100.0	0.0	20.3 ± 0.6	14.4 ± 0.6	4.1 ± 0.1	9.2 ± 0.5	96.3 ± 3.3	18.7 ± 1.3	24.8 ± 2.0	6.1 ± 1.0
		M	2,682 ± 221	2,039 ± 181	609 ± 79	86.7	13.3	21.4 ± 0.9	15.0 ± 0.8	4.3 ± 0.2	9.7 ± 0.9	96.4 ± 5.2	20.8 ± 2.1	27.5 ± 3.4	6.8 ± 1.5
	SBY	F	2,561 ± 119	1,929 ± 92	553 ± 47	100.0	0.0	22.0 ± 1.0	14.3 ± 0.5	4.4 ± 0.2	9.1 ± 0.6	91.0 ± 5.1	20.0 ± 1.4	25.9 ± 2.3	5.9 ± 1.3
		M	2,948 ± 223	2,234 ± 157	629 ± 79	90.0	10.0	23.2 ± 1.0	15.1 ± 0.6	4.6 ± 0.2	9.3 ± 0.8	89.7 ± 6.2	21.6 ± 1.7	28.0 ± 2.5	6.4 ± 1.3
7	HBY	F	2,996 ± 229	2,325 ± 189	730 ± 90	91.7	8.3	22.6 ± 0.9	15.9 ± 0.8	4.5 ± 0.2	10.5 ± 0.7	97.6 ± 3.9	23.8 ± 1.9	32.1 ± 2.8	8.3 ± 1.5
		M	3,483 ± 266	2,692 ± 215	816 ± 92	78.3	21.7	23.7 ± 0.9	16.7 ± 0.7	4.7 ± 0.2	10.7 ± 1.0	95.9 ± 5.6	25.2 ± 2.2	34.3 ± 3.9	9.1 ± 2.0
	SBY	F	3,135 ± 194	2,417 ± 163	689 ± 82	96.7	3.3	23.5 ± 0.9	15.5 ± 0.6	4.7 ± 0.2	10.1 ± 0.7	93.2 ± 4.0	23.8 ± 2.1	31.4 ± 2.8	7.6 ± 1.2
		M	3,477 ± 290	2,675 ± 237	754 ± 109	90.0	10.0	24.3 ± 0.8	16.3 ± 0.8	4.9 ± 0.2	10.2 ± 0.8	91.7 ± 4.9	24.8 ± 2.0	31.6 ± 3.2	6.8 ± 1.7
8	HBY	F	3,573 ± 262	2,823 ± 210	916 ± 94	95.0	5.0	23.1 ± 0.9	16.3 ± 0.6	4.6 ± 0.2	10.7 ± 0.7	98.0 ± 3.7	24.7 ± 1.9	33.1 ± 3.1	8.4 ± 1.6
		M	4,160 ± 319	3,265 ± 270	1,029 ± 135	61.7	38.3	24.6 ± 0.9	17.7 ± 0.8	4.9 ± 0.2	11.5 ± 1.1	98.2 ± 5.2	28.2 ± 2.9	38.3 ± 5.0	10.1 ± 2.5
	SBY	F	3,730 ± 200	2,930 ± 156	906 ± 58	100.0	0.0	23.9 ± 0.8	16.3 ± 0.6	4.8 ± 0.2	10.4 ± 0.7	94.2 ± 4.2	24.9 ± 1.9	32.5 ± 3.0	7.6 ± 1.4
		M	4,164 ± 292	3,246 ± 235	948 ± 114	86.7	13.3	25.8 ± 0.9	17.1 ± 0.9	5.2 ± 0.2	10.8 ± 1.1	92.6 ± 6.1	27.9 ± 2.7	36.8 ± 4.7	8.9 ± 2.6
9	HBY	F	3,886 ± 246	3,084 ± 214	980 ± 114	81.7	18.3	25.6 ± 1.0	18.1 ± 0.9	5.1 ± 0.2	11.7 ± 0.9	97.5 ± 4.9	29.9 ± 2.4	40.3 ± 4.0	10.4 ± 2.3
		M	4,430 ± 374	3,554 ± 314	1,047 ± 147	63.3	36.7	26.1 ± 1.0	18.3 ± 0.9	5.2 ± 0.2	11.8 ± 1.1	96.2 ± 5.2	30.8 ± 3.0	41.4 ± 4.8	10.6 ± 2.4
	SBY	F	4,058 ± 318	3,123 ± 210	956 ± 98	90.0	10.0	26.0 ± 0.9	17.7 ± 0.5	5.2 ± 0.2	11.3 ± 0.8	94.1 ± 4.9	29.4 ± 1.8	38.9 ± 3.1	9.5 ± 2.0
		M	4,628 ± 435	3,635 ± 358	1,055 ± 138	83.3	16.7	27.3 ± 1.1	18.4 ± 0.8	5.5 ± 0.2	11.5 ± 1.0	92.7 ± 6.2	31.3 ± 2.5	40.8 ± 4.1	9.5 ± 1.9
10	HBY	F	4,296 ± 208	3,429 ± 176	1,124 ± 99	78.3	21.7	25.5 ± 0.9	18.4 ± 0.9	5.1 ± 0.2	12.1 ± 0.9	99.3 ± 4.2	31.0 ± 2.6	40.7 ± 3.8	9.8 ± 2.0
		M	5,121 ± 417	4,103 ± 343	1,306 ± 149	58.3	41.7	26.5 ± 1.2	19.2 ± 0.9	5.3 ± 0.2	13.0 ± 1.3	101.1 ± 5.6	34.6 ± 3.8	47.1 ± 6.1	12.6 ± 3.1
	SBY	F	4,252 ± 244	3,344 ± 207	1,014 ± 93	90.0	10.0	26.4 ± 0.9	17.9 ± 0.7	5.3 ± 0.2	11.7 ± 0.9	95.3 ± 4.2	30.9 ± 2.7	39.9 ± 3.5	9.0 ± 2.0
		M	4,963 ± 290	3,951 ± 241	1,178 ± 152	80.0	20.0	27.1 ± 1.2	18.7 ± 0.9	5.4 ± 0.2	12.3 ± 1.3	96.5 ± 6.2	33.3 ± 4.0	45.0 ± 6.7	11.7 ± 3.5

1 Measurements are expressed as the mean ± SD [n = 60 for high breast-yielding (HBY) birds by sex and age, and n = 30 for standard breast-yielding (SBY) birds by sex and age].

2 F = female; M = male.

3 WOG = carcass without giblets.

4 No = normal or mildly affected fillets by WB condition; Yes = fillets moderately or severely affected by WB condition.

5 Parameters for carcass conformation: M0 = breast length; M1 = breast width in the cranial region; M2 = a vertical line from the tip of keel to 1/5th of breast length; M3 = breast width at the end of M2; M4 = angle formed at the tip of keel and extending to outer points of M3; M5 = area of the triangle formed by M3 and lines generated by M4; M6 = area of the breast above M3; M7 = difference of areas M6 minus M5.

### Image Collection

The image acquisition of broiler carcasses was performed before evisceration using a black background and a ruler that was placed vertically on the background to define the spatial scale of each image when determining carcass measurements. A Canon EOS 60D digital single-lens reflex camera (Canon USA Inc., Lake Success, NY) was used to capture images. The orientation of the carcass hanging vertically down was such that its breast section was facing the camera. Indeed, this device was placed centrally in front of the carcass at 1.32 m of distance from the camera lens to the shackle line. To collect broiler carcass images, settings of 2,592 × 1,728 pixels and spatial resolution of 72 ppi were used. Images were captured with an exposure time of 1/60 s and opening f/5 with a format of JPG image file.

### Deboning and Tactile Evaluation

Broiler carcasses were manually hot-deboned by severing the humeral-scapular joint and pulling firmly downward on the wings at 30 min postmortem in a 10°C to 13°C processing room. Whole breast fillets were scored approximately 30 min after deboning (i.e., 1 h postmortem) for degree of hardness by palpation assessment as described by Tijare et al. (2016). Trained personnel scored all fillets and have substantial experience scoring hot deboned and chilled deboned fillets. As per our previous results (Caldas-Cueva et al., 2021), the scored fillets were categorized into 2 groups (binary response): negative response (No) = unaffected fillets or fillets mildly affected by WB condition (WB scores 0.0, 0.5, 1.0, or 1.5), and positive response (Yes) = fillets moderately or severely affected by WB condition (WB scores 2.0, 2.5 or 3.0). The people scoring fillets had a minimum of 3 yr of scoring fillets for WB that were deboned before (hot-deboning) or after chilling. Weights of broiler breast fillets along with WB incidence rates across strains, gender, and ages are also shown in Table 1.

### Image Processing and Analysis

**Figure 1:**
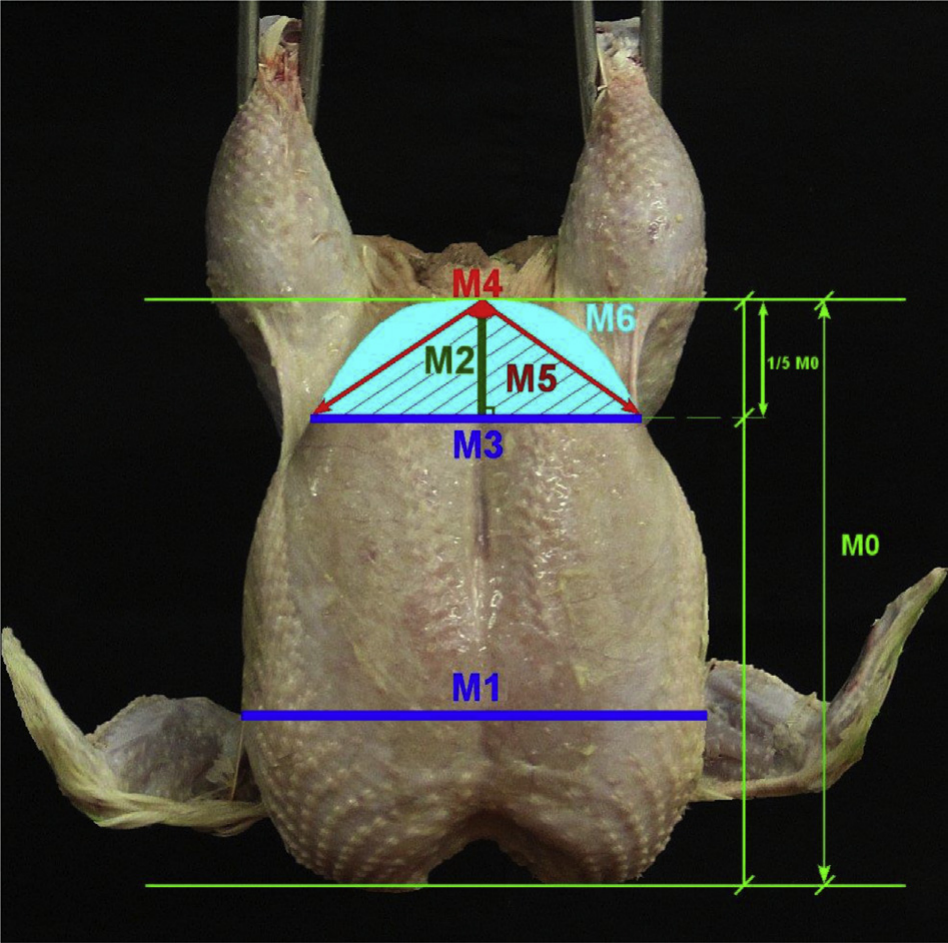
Conformational measurements from broiler carcass images (Caldas-Cueva et al., [2021]). Parameters for carcass conformation: M0 = breast length; M1 = breast width in the cranial region; M2 = a vertical line from the tip of keel to 1/5th of breast length; M3 = breast width at the end of M2; M4 = angle formed at the tip of keel and extending to outer points of M3; M5 = area of the triangle formed by M3 and lines generated by M4; M6 = area of the breast above M3.

Images of broiler carcasses were processed using the ImageJ software (National Institutes of Health, Bethesda, MD). Image processing functions such as vertical rotation and sharpening were used. The conformational measurements obtained from broiler carcass images were those considered in our previous experiments as shown in Figure 1 (Caldas-Cueva et al., 2021). Briefly, M0 was the breast length, M1 was the breast width at the widest part of the cranial section, M2 was 1/5th of M0 starting from the tip of keel, M3 was the breast width in the caudal region which was taken at the end of M2, M4 was the angle formed at the tip of keel and extending to outer points of M3, M5 was the triangle area formed by M3 and lines generated by M4, M6 was the breast area above M3, and M7 was M6 minus M5. Furthermore, 4 ratios [(ratio of M3 to M1 (M8), ratio of M3 to M2 (M9), ratio of M7 to M5 (M10), and ratio of M1 to M0 (M11)] were calculated and included in the analysis. Image measurements from broiler carcasses across strains, gender, and ages are also shown in Table 1.

### Statistical Analysis

Spearman's correlation coefficients (*r*_*s*_) were estimated for WB tactile scores and image measurements. A stratified random split was used to divide the data into 2 sets of 70 and 30% for training and validation, respectively. A binary response or WB condition status (Yes/No) was considered in this experiment because our previous results suggest that it was the most suitable approach to develop prediction models using measurements from broiler carcass images (Caldas-Cueva et al., 2021). Thus, the binary logistic regression platform was performed to assess and select suitable prediction models with a binomial distribution for WB condition status. The predicting quality or classification performance of prediction models were evaluated based on the statistical outputs considered in our previous experiments (Caldas-Cueva et al., 2021). Briefly, the overall misclassification rate (MR = 1– overall accuracy), the true positive rate or sensitivity (**ST**), false positive rate (FPR = 1 – specificity), and area under the receiver operating characteristic curve or AUC. Moreover, generalized (Gen.) R^2^, root mean square error, and mean absolute deviation (**MAD**) were also considered. Once the best model was selected, the binary logistic regression analysis was run by broiler strain (HBY and SBY strains), sex (female, male, and as-hatched), age (from 6–10 wk), live weight [< 3.402 kg (7.5 lb) and ≥3.402 kg (7.5 lb)], and broiler strain across live weight. The statistical analysis was achieved using JMP software, version 14.1.0 (SAS Institute Inc., Cary, NC).

## Results and discussion

### Woody Breast Scores and Measurements From Broiler Carcass Images

**Figure 2:**
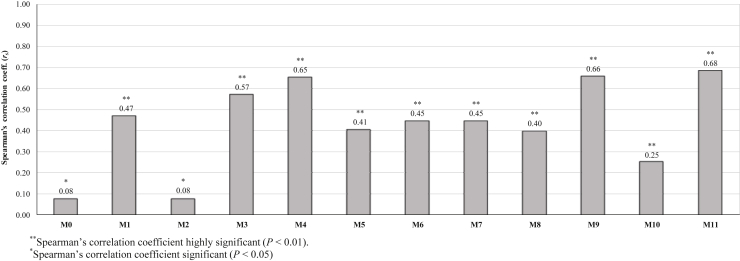
Correlations of image measurements with woody breast (WB) tactile scores. ∗∗Spearman's correlation coefficient highly significant (*P* < 0.01). ∗Spearman's correlation coefficient significant (*P* < 0.05).

Spearman's correlation coefficients (*r*_*s*_) between WB tactile scores and conformational measurements obtained from broiler carcass images are shown in Figure 2. Overall, ratios M11 (M1/M0, *r*_*s*_ = 0.68) and M9 (M3/M2, *r*_*s*_ = 0.66) as well as the image measurement M4 (angle at keel, *r*_*s*_ = 0.65) had the highest correlation to WB score (*P* < 0.01), whereas measurements M3, M1, M7, M6, M5, and M8 presented a moderate correlation (0.40 ≤ *r*_*s*_ ≤ 0.57; *P* < 0.01). On the other hand, the ratio M10 (M7/M5), and measurements M2 and M0 showed low and very weak correlations to WB score (*r*_*s*_ ≤ 0.25; *P* < 0.05), respectively. The highest correlation coefficients to WB scores showed by M11, M9, and M4 measurements were comparable with those observed in our previous experiments (*r*_*s*_ ≥ 0.70; *P* < 0.01), which additionally highlighted that the same measurements including M3 presented the highest correlation coefficients to compression force (*r*_*s*_ ≥ 0.64; *P* < 0.01) (Caldas-Cueva et al., 2021). These relationships support the hypothesis that the breast conformation of broiler carcasses undergoes a width increase as WB severity increases, which may be consistent with findings of previous studies (Zambonelli et al., 2016; Griffin et al., 2018) that reported a significant increase in breast width of fillets affected by WB and white striping (**WS**) defects compared to normal samples. However, Griffin et al. (2018) reported that the length and width of breast fillets as predictors in a cumulative logit proportional odds model were inversely related to the WB severity, even though they also observed that increasing the *P. minor* width, *P. major* depth, and *P. major* yield increased the odds of obtaining a muscle with an increased progression and WB severity. Regardless of these contrasting reports about the morphometric measurement changes due to WB condition in broiler breast fillets, the results from this study suggest an evident relationship between WB severity and broiler carcass features, which provides a basis for further assessment of the use of these image measurements for predicting the WB condition.

### Binary Logistic Regression Models for Predicting WB Condition

Morphometric changes from live broilers or broiler carcasses measured directly or through image analysis have been used in other studies to predict important indicators such as chemical composition of broiler carcasses (Chambers and Fortin, 1984), body weight (De Wet et al., 2003; Mollah et al., 2010), and rigor mortis development in broiler muscles (Cavitt and Sams, 2003). In this sense, the 8 measurements from broiler carcass images (M0 through M7) and 4 ratios (M8 through M11) were used as predictor variables in the statistical analysis to develop and select suitable models for predicting WB condition. The results from the binary logistic regression analysis showed 6 prediction models (Table 2) that were the most acceptable models for predicting WB condition based on their predictive performance outcomes. The binary logistic regression results of this study were similar to those observed in our previous experiments, which also presented that the most satisfactory prediction models for WB detection in broiler carcasses were based on carcass measurements M1, M2, M3, M4, and M6 as well as ratios M9 and M11 (Caldas-Cueva et al., 2021).

**Table 2:** Binary logistic regression models to predict woody breast (WB) condition in broiler carcasses using image measurements.

Prediction model^1^	Data set^2^	Fit details							Parameter estimates and odds ratio (OR)				
		Gen. R^2^	RMSE	MAD	MR (%)	TPR (%)	FPR (%)	AUC	Parameter	Estimate	Std. Error	Pr > χ2	OR
Model 1	Training	0.65	0.26	0.13	9.05	63.21	3.44	0.95	M1	1.39	0.30	<0.0001	4.03
	Validation	0.61	0.26	0.13	8.52	71.11	4.44	0.94	M2	−7.55	1.00	<0.0001	5.24 × 10^−4^
									M3	1.83	0.28	<0.0001	6.23
Model 2	Training	0.62	0.27	0.14	9.84	60.38	3.82	0.95	M1	1.68	0.29	<0.0001	5.38
	Validation	0.58	0.27	0.15	9.63	64.44	4.44	0.93	M2	−4.40	0.99	<0.0001	1.23 × 10^−2^
									M4	0.33	0.06	<0.0001	1.38
Model 3	Training	0.62	0.26	0.14	10.00	60.38	4.01	0.95	M1	1.76	0.28	<0.0001	5.82
	Validation	0.58	0.27	0.14	8.52	68.89	4.00	0.93	M2	−9.45	1.06	<0.0001	7.91 × 10^−5^
									M6	0.31	0.06	<0.0001	1.37
Model 4	Training	0.60	0.27	0.14	10.00	56.60	3.24	0.94	M9	8.86	1.30	<0.0001	7.07 × 10^3^
	Validation	0.58	0.27	0.15	10.00	57.78	3.56	0.93	M11	26.28	6.55	<0.0001	2.60 × 10^11^
Model 5	Training	0.63	0.26	0.14	10.79	58.49	4.58	0.95	M1	0.63	0.11	<0.0001	1.87
	Validation	0.56	0.27	0.14	9.26	66.67	4.44	0.92	M9	11.10	1.22	<0.0001	6.62 × 10^4^
Model 6	Training	0.63	0.26	0.14	10.00	60.38	4.01	0.95	M3	1.09	0.15	<0.0001	2.99
	Validation	0.61	0.26	0.14	8.52	73.33	4.89	0.94	M11	48.08	6.31	<0.0001	7.59 × 10^20^

M1, M2, …, Mn are the independent predictor variables (image measurements), α is the intercept, and β1, β2, …, βn are the regression coefficients.

Abbreviations: AUC, area under the ROC (receiver operating characteristic) curve; FPR, false positive rate; MAD, mean absolute deviation; MR, misclassification rate; RMSE, root mean square error; TPR, true positive rate (sensitivity–ST)

1 Model 1 = Logit (*p*) = α + β1 M1 + β2 M2 + β3 M3. Model 2 = Logit (*p*) = α + β1 M1 + β2 M2 + β3 M4. Model 3 = Logit (*p*) = α + β1 M1 + β2 M2 + β3 M6. Model 4 = Logit (*p*) = α + β1 M9 + β2 M11. Model 5 = Logit (*p*) = α + β1 M1 + β2 M9. Model 6 = Logit (*p*) = α + β1 M3 + β2 M11.

2 Training {n = 630: No [WB scores 0.0 or 0.5 (n = 376), and 1.0 or 1.5 (n = 148)], and Yes [WB scores 2.0 or 2.5 (n = 76), and 3.0 (n = 30)]} and validation {n = 270: No [WB scores 0.0 or 0.5 (n = 160), and 1.0 or 1.5 (n = 65)], and Yes [WB scores 2.0 or 2.5 (n = 35), and 3.0 (n = 10)]}.

Regardless of the training and validation sets, established binary regression models showed an overall accuracy [(1 – MR)∗100% = the percentage of broiler carcasses that were correctly classified as WB or normal] greater than 89% with an ST higher than 56%, which indicates the proportion of broiler carcasses that were correctly classified as affected samples with WB condition, and the proportion of broiler carcasses that were incorrectly identified as affected samples with WB condition was lower than 5%. In addition, the measure of how well the models can differentiate between 2 levels of the binary response was excellent (AUC ≥ 0.92) with a Gen. R^2^ greater than or equal to 0.56, and root mean square error and MAD values less than or equal to 0.27 and 0.15, respectively. Among these prediction models for WB condition, the results from the binary logistic regression analysis of the training set showed that the model 1 had the lowest MR (9.05%) and the highest ST (63.21%) and Gen. R^2^ (0.65) as well the lowest MAD value (0.13). When validating the models, the lowest levels of MR (≤9%) and the highest levels of ST (≥68%) were presented by models 1, 3, and 6. In addition, all models also displayed low FPR levels (≤5%) and high AUC values (≥0.92). These results suggested that the model 1 that included M1, M2, and M3 measurements had the best classification performance for predicting WB condition in broiler carcasses; therefore, this model was selected for further binary logistic regression analyses by broiler strain, sex, age, live weight, and strain across live weight at slaughter. These outcomes are supported by the conclusions of our previous experiments, which also indicated that the simplest validated prediction model for WB condition included M1, M2, and M3 measurements (Caldas-Cueva et al., 2021).

Table 2 also shows the strength of logistic relationships between the binary response (Yes/No) and predictor variables which was observed in the corresponding odds ratios for each binary logistic regression model. Analyzing the odds ratios for the model 1, the odds of detecting an affected sample with WB condition were 4.03 and 6.23 times higher with 1-cm increase in the breast width at the cranial (M1) and caudal (M3) regions of broiler carcasses, respectively. Although there are conformational changes in the breast muscle intrinsic to the selection of broilers for rapid growth and greater yields (Godfrey and Goodman, 1956; Reddish and Lilburn, 2004; Griffin et al., 2018), the significant increase in physical dimensions observed in breast fillets with WB myopathy (Mudalal et al., 2015; Zambonelli et al., 2016; Kuttappan et al., 2017) could help to explain the abnormal increase of the breast width at cranial (M1) and caudal (M3) sections of broiler carcasses with WB condition compared with normal carcasses.

On the other hand, the odds of observing an affected sample with WB condition became 99.95% smaller with 1-cm increase in the 1/5th of the breast length (M2) of broiler carcasses. This result is in agreement with the findings of Griffin et al. (2018), who reported that the breast fillet length as a predictor was negatively related to the WB severity, which means that the longer the breast length, the lower the WB severity. Moreover, evaluating the odds ratios for the other image measurements in the model 2 (M4) and model 3 (M6), the odds of obtaining an affected sample with WB myopathy increased by 38.40 and 36.77% with one-degree increase in the angle at keel tip (M4) and one square centimeter increase in the breast area at the caudal region (M6) of broiler carcasses respectively. As per these results, morphometric changes were more evident at the caudal region of the breast in broiler carcasses with severe levels of WB condition. These findings can be tentatively explained by the fact that broiler breast fillets with severe degrees of WB myopathy exhibit changes in shape that includes a ridge-like protuberance at the caudal section (Sihvo et al., 2014; Mudalal et al., 2015; Griffin et al., 2018).

### Model Performance Evaluation by Broiler Strain

Table 3 shows a summary of the predictive performance of the selected binary logistic regression model for detecting WB condition in broiler carcasses by strain. Regardless of the broiler sex and age or live weight, the HBY strain showed a higher ST (68.54 and 73.53% for training and validation, respectively) than the SBY strain (60.00 and 62.50% for training and validation, respectively); however, the SBY strain presented a slightly lower MR (5.71 and 5.56% for training and validation, respectively) in comparison with HBY strain (9.76 and 8.89% for training and validation, respectively). These differences in the predictive performance could be associated with the genotype factor due to there are differences in the development and degree of WB severity between broiler strains. There are studies reporting that HBY hybrid showed higher degrees of myopathic abnormalities coupled with an impaired meat quality compared with the SBY strain (Petracci et al., 2013; Mazzoni et al., 2015). Thus, broilers from HBY strain may exhibit more conformational changes in their carcass breast region that would result in higher levels of ST, whereas the higher levels of accuracy found for SBY strain may be attributed to the fact that the model correctly classified mostly normal samples because SBY broilers showed lower overall WB incidence and severity rates.

**Table 3:** Predictive performance of the selected binary logistic regression model for detecting woody breast (WB) condition in broiler carcasses by strain using image measurements.

Strain	Data set^1^	Fit details^2^							Parameter estimates and odds ratio (OR)				
		Gen. R^2^	RMSE	MAD	MR (%)	TPR (%)	FPR (%)	AUC	Parameter	Estimate	Std. Error	Pr > χ2	OR
High breast-yielding (HBY)	Training	0.65	0.28	0.15	9.76	68.54	3.93	0.95	M1	1.53	0.35	<0.0001	4.64
	Validation	0.62	0.27	0.15	8.89	73.53	4.79	0.93	M2	−8.03	1.19	<0.0001	3.24 × 10^−4^
									M3	1.84	0.31	<0.0001	6.30
Standard breast-yielding (SBY)	Training	0.59	0.21	0.09	5.71	60.00	2.11	0.96	M1	1.37	0.62	0.0277	3.95
	Validation	0.65	0.19	0.08	5.56	62.50	2.44	0.98	M2	−7.95	2.12	0.0002	3.53 × 10^−4^
									M3	1.84	0.50	0.0003	6.31

Abbreviations: AUC, area under the ROC (receiver operating characteristic) curve; FPR, false positive rate; MAD, mean absolute deviation; MR, misclassification rate; RMSE, root mean square error; TPR, true positive rate (sensitivity, ST).

1 Training (HBY: n = 420; SBY: n = 210) and validation (HBY: n = 180; SBY: n = 90).

2 The model used for WB prediction (Model 1): Logit (p) = α + β1 M1 + β2 M2 + β3 M3.

On the other hand, both strains presented low FPR levels (<5%) or high percentages of specificity [(1 – FPR)∗100%] (>95%), which was estimated as the ratio of the number of broiler carcasses that were correctly classified as negative to the total number of negatives or normal and mildly affected samples. In addition, the model displayed an excellent ability to distinguish the 2 levels of the binary response for both strains (AUC ≥ 0.93). Analyzing the odds ratio for each predictor variable, the odds of observing an affected sample with WB myopathy were 4.64 and 3.95 times higher with 1-cm increase in the measurement M1, whereas the odds were 6.30 and 6.31 times higher with 1-cm increase in the measurement M3 of broiler carcasses from the HBY and SBY strains, respectively. By contrast, the odds of observing an affected sample with WB condition became on average 99.97% smaller with 1-cm increase in the measurement M2 of broiler carcasses of both strains.

### Model Performance Evaluation by Broiler Sex

To evaluate the effect of broiler sex on the predictive performance, the binary logistic regression analysis was carried out by gender using the selected model. Table 4 shows the results of this analysis for female, male, and as-hatched broilers. In female broilers, the MR (4.44%) and FPR (1.75%) levels were lower than those for male broilers (13.65 and 8.82% for MR and FPR, respectively). These differences were more noticeable when analyzing the validation set for both genders. However, the ST for male broilers were higher in training (71.43%) and validation (74.29%) sets than that for female broilers (68.97 and 60.00% for training and validation, respectively), which indicates that the model in male broilers has a better performance in terms of the proportion of broilers carcasses that were correctly classified as affected samples among the total number of positives or WB samples. Performance differences in terms of accuracy and sensitivity of the prediction model could be related to contrasts in the occurrence and severity of WB myopathy between female and male broilers. Indeed, there are reports confirming that male broilers showed a higher WB incidence and severity with differentiated biological features that could make them more prone to WB condition than female broilers (Trocino et al., 2015; Brothers et al., 2019). In this experiment, the overall occurrence of moderate to severe cases of WB myopathy in male broilers was approximately 3 times higher compared to female broilers. Therefore, male broilers carcasses could exhibit more detectable conformational changes that would result in higher levels of ST, whereas the lower incidence and severity of WB condition in female broiler carcasses could explain the higher levels of accuracy (>95%) because the model correctly classified mostly normal carcasses that was reflected in high levels of AUC (≥0.97).

**Table 4:** Predictive performance of the selected binary logistic regression model for detecting woody breast (WB) condition in broiler carcasses by sex using image measurements.

Sex	Data set^1^	Fit details^2^							Parameter estimates and odds ratio (OR)				
		Gen. R^2^	RMSE	MAD	MR (%)	TPR (%)	FPR (%)	AUC	Parameter	Estimate	Std. Error	Pr > χ^2^	OR
Female	Training	0.69	0.18	0.07	4.44	68.97	1.75	0.97	M1	2.07	0.58	0.0003	7.94
	Validation	0.65	0.18	0.07	4.44	60.00	1.60	0.98	M2	−9.91	2.14	<0.0001	4.99 × 10^−5^
									M3	2.30	0.64	0.0003	10.02
Male	Training	0.64	0.30	0.18	13.65	71.43	8.82	0.93	M1	1.47	0.37	<0.0001	4.37
	Validation	0.63	0.31	0.17	14.81	74.29	11.00	0.93	M2	−8.03	1.21	<0.0001	3.25 × 10^−4^
									M3	1.36	0.28	<0.0001	3.90
As-hatched	Training	0.65	0.26	0.13	9.05	63.21	3.44	0.95	M1	1.39	0.30	<0.0001	4.03
	Validation	0.61	0.26	0.13	8.52	71.11	4.44	0.94	M2	−7.55	1.00	<0.0001	5.24 × 10^−4^
									M3	1.83	0.28	<0.0001	6.23

Abbreviations: AUC, area under the ROC (receiver operating characteristic) curve; FPR, false positive rate; MR, misclassification rate; MAD, mean absolute deviation; RMSE, root mean square error; TPR, true positive rate (sensitivity, ST).

1 Training (n = 315) and validation (n = 135). As-hatched group included all data set (n = 900) divided into 2 sets of training (n = 630) and validation (n = 270).

2 The model used for WB prediction (model 1): Logit (p) = α + β1 M1 + β2 M2 + β3 M3.

Table 4 also shows the results of the binary logistic regression analysis using all data (50% females and 50% males), which was classified as as-hatched broilers in this experiment. Intermediate validated levels of MR (8.52%), true positive rate or ST (71.11%), FPR (4.44%), and AUC (0.94) was found for as-hatched broilers, which indicate that the predictive performance of the model using all data was between those presented by female and male broilers. With respect to the odds ratio for each predictor variable, an increase of 1 cm in the measurement M1 yielded similar odds of detecting an affected sample with WB condition (on average 4.20) in male and as-hatched broilers, whereas an increase of 1-cm in the same dimension generated higher odds of obtaining an affected sample (7.94) in female broilers. Moreover, the odds were 10.02, 3.90, and 6.23 times higher with 1-cm increase in the measurement M3 of female, male, and as-hatched broilers, respectively. By contrast, the odds of observing an affected sample with WB condition became on average 99.97% smaller with 1-cm increase in the measurement M2 of all broiler carcasses.

### Model Performance Evaluation by Broiler Age

The results of the binary logistic regression analysis by age are shown in Table 5. Regardless of the broiler strain and sex, the best-fit model for WB prediction in broiler carcasses from 6 and 7 wk included the image measurement M1 and ratio M9 (Gen. R^2^ ≥ 0.52, MR ≤ 7.41%, and ST ≥ 50%), whereas the model for broilers from 8 to 10 wk included image measurements M1, M2, and M3 (Gen. R^2^ ≥ 0.54, MR ≤ 18.52%, and ST ≥ 62.50%). Regardless of the training and validation sets, the MR levels increased as broiler age increased; however, these values were less than 20%. In the training set, the highest levels of ST were found at 8 (75%) and 9 (74.07%) weeks of age, whereas the lowest ST level was found at the youngest age (6 wk, 57.14%). Although the ST values were lower in the validation set, similar patterns were observed. With regard to FPR and AUC parameters, FPR levels were lower than 6% and AUC values greater than or equal to 0.95 for broiler carcasses from 6 to 9 wk of age, whereas FPR levels for 10-week-old broiler carcasses were higher than 6% and AUC values between good (0.89, training) and excellent (0.94, validation). As expected, higher levels of WB occurrence were found in older broilers that could explain differences in the predictive performance among broilers from different ages. It has been reported high levels of incidence and degrees of WB severity in older broilers (Kuttappan et al., 2017), which is consistent with another study showing that the occurrence of broilers with muscle fiber degeneration increases with age (Radaelli et al., 2017). Validated levels of ST increased from 6 to 8 wk; however, this parameter started to decrease at 9 wk. Thus, high levels of WB frequency did not result necessarily in high levels of ST because broiler carcasses from 10 wk of age presenting the highest WB occurrence rate showed ST levels lower than those for 8- and 9-week-old broilers. On the other hand, the highest level of accuracy found in the youngest group of broiler carcasses (6 wk) could be associated with the fact that the model properly sorted mostly normal carcasses because these birds had the lowest overall WB incidence and severity rates. In addition, it is important to highlight that validated levels of FPR were low with values less than or equal to 5% for most broiler carcasses (6–9 wk).

**Table 5:** Predictive performance of selected binary logistic regression models for detecting woody breast (WB) condition in broiler carcasses by age using image measurements.

Age (wk)	Live weight^1^ (g)	Data set^2^	Fit details^3^							Parameter estimates and odds ratio (OR)				
			Gen. R^2^	RMSE	MAD	MR (%)	TPR (%)	FPR (%)	AUC	Parameter	Estimate	Std. Error	Pr > χ2	OR
6	2,602 ± 21	Training	0.63	0.16	0.05	3.97	57.14	1.68	0.97	M1	2.27	1.10	0.0385	9.72
		Validation	0.58	0.20	0.07	5.56	50.00	2.00	0.96	M9	11.20	4.44	0.0116	7.33 × 10^4^
7	3,262 ± 25	Training	0.65	0.23	0.10	7.14	66.67	3.60	0.96	M1	1.27	0.62	0.0412	3.57
		Validation	0.52	0.25	0.09	7.41	57.14	2.13	0.95	M9	12.51	3.28	0.0001	2.70 × 10^5^
8	3,893 ± 29	Training	0.78	0.20	0.08	6.35	75.00	2.83	0.98	M1	3.68	1.29	0.0044	0.40 × 10^2^
		Validation	0.63	0.28	0.10	9.26	70.00	4.55	0.96	M2	−9.56	3.76	0.0110	7.05 × 10^−5^
										M3	2.61	0.92	0.0043	13.64
9	4,220 ± 33	Training	0.74	0.25	0.12	9.52	74.07	5.05	0.97	M1	2.02	0.77	0.0088	7.55
		Validation	0.76	0.27	0.13	12.96	64.29	5.00	0.98	M2	−7.32	2.62	0.0052	6.59 × 10^−4^
										M3	2.48	0.84	0.0031	11.94
10	4,675 ± 38	Training	0.54	0.32	0.21	13.49	64.52	6.32	0.89	M1	1.10	0.45	0.0144	2.99
		Validation	0.64	0.32	0.21	18.52	62.50	10.53	0.94	M2	−6.45	1.76	0.0003	1.57 × 10^−3^
										M3	1.45	0.38	0.0001	4.26

Abbreviations: AUC, area under the ROC (receiver operating characteristic) curve; FPR, false positive rate; MAD, mean absolute deviation; MR, misclassification rate; RMSE, root mean square error; TPR, true positive rate (sensitivity, ST).

1 Means ± SEM.

2 Training (n = 126) and validation (n = 54).

3 The models used for WB prediction. Model 1: Logit (p) = α + β1 M1 + β2 M2 + β3 M3 (8 – 10 wk). Model 5: Logit (p) = α + β1 M1 + β2 M9 (6 and 7 wk).

Analyzing the odds ratio for each predictor variable, an increase of 1 cm in the measurement M1 yielded the highest odds of detecting an affected sample with WB condition in 8-week-old broilers (0.40 × 10^2^) followed by 6-, 9-, 7-, and 10-week-old broilers, respectively. Moreover, an increase of 1 cm in the measurement M3 also produced variable odds of obtaining an affected sample in broiler carcasses from 8 (13.64), 9 (11.94), and 10 (4.26) weeks of age. Conversely, the odds of observing a carcass affected by WB condition became on average 99.92% smaller with 1-cm increase in the measurement M2 of broiler carcasses from 8 to 10 wk of age. Because one of the predictors of the model was a ratio (M9), one-unit increase in this ratio yielded considerably high odds ratios of detecting a carcass affected by WB in broiler carcasses from 6 (7.33 × 10^4^) and 7 (2.70 × 10^5^) weeks of age.

### Model Performance Evaluation by Broiler Live Weight

Predictive performance parameters as well as parameter estimates and odds ratios for broiler carcasses divided by live weight at slaughter (<3.402 kg and ≥3.402 kg) are reported in Table 6. The results in the training and validation sets showed that lighter birds (<3.402 kg) had lower levels of MR (<6%) and FPR (<4%) in comparison with heavier broilers. However, the ST levels were higher for heavier broilers (>67%) compared with lighter birds, which indicates that the model in heavier broilers showed a better performance in terms of the proportion of broilers carcasses that were correctly classified as positive among the total number of carcasses moderately or severely affected by WB defect. Similar to male, older, or HBY broilers, these higher rates of ST observed in heavier broilers could be associated with higher overall proportions of WB occurrence and severity found in this group of birds, which is in agreement with Cruz et al. (2017) who reported that heavier broilers presented higher rates of WB incidence and severity. Moreover, lower levels of MR could be explained by a lower WB frequency observed in lighter birds, which indicates that the model properly classified mainly normal carcasses. On the other hand, the ability to distinguish the 2 levels of the binary response was excellent (AUC ≥ 0.93) for both groups.

**Table 6:** Predictive performance of the selected binary logistic regression model for detecting woody breast (WB) condition in broiler carcasses by live weight using image measurements.

Live weight (kg)	Data set^1^	Fit details^2^							Parameter estimates and odds ratio (OR)				
		Gen. R^2^	RMSE	MAD	MR (%)	TPR (%)	FPR (%)	AUC	Parameter	Estimate	Std. Error	Pr > χ2	OR
<3.402 (<7.5 lb)	Training	0.63	0.19	0.07	4.00	55.56	0.48	0.96	M1	1.68	0.78	0.0317	5.34
	Validation	0.53	0.18	0.06	5.15	60.00	3.26	0.98	M2	−7.49	2.46	0.0023	5.57 × 10^−4^
									M3	2.66	0.81	0.0010	14.26
≥3.402 (≥7.5 lb)	Training	0.60	0.30	0.17	10.62	67.05	4.42	0.93	M1	1.39	0.31	<0.0001	4.03
	Validation	0.68	0.28	0.17	11.56	70.00	6.02	0.95	M2	−7.33	1.09	<0.0001	6.53 × 10^−4^
									M3	1.43	0.26	<0.0001	4.18

Abbreviations: AUC, area under the ROC (receiver operating characteristic) curve; FPR, false positive rate; MAD, mean absolute deviation; MR, misclassification rate; RMSE, root mean square error; TPR, true positive rate (sensitivity, ST).

1 Training (<3.402 kg: n = 225; ≥3.402 kg: n = 405) and validation (<3.402 kg: n = 97; ≥3.402 kg: n = 173).

2 The model used for WB prediction (model 1): Logit (p) = α + β1 M1 + β2 M2 + β3 M3.

The strength of logistic relationships between the binary response and predictor variables for both lighter and heavier broilers was observed in the corresponding odds ratios for each binary logistic regression output (Table 6). The odds of detecting a carcass affected by WB myopathy were 5.34 and 4.03 times higher with 1-cm increase in the measurement M1, whereas the odds were 14.26 and 4.18 times higher with 1-cm increase in the measurement M3 of broiler carcasses corresponding to lighter and heavier birds, respectively. By contrast, the odds of detecting an affected sample with WB condition was reduced on average by 99.94% with 1-cm increase in the M2 measurement of broiler carcasses of both groups.

To further evaluate the capacity of the selected model, predictive performance parameters and other statistical outputs from binary logistic regression analysis performed based on broiler strain at different live weights are presented in Table 7. Regardless of the data set and within the HBY strain group, lighter broilers had a high predictive performance in terms of overall accuracy and specificity because these birds presented lower levels of MR (<5%) and FPR (<2%) compared with those for heavier birds (MR > 11% and FPR > 6%). However, heavier birds showed a higher predictive performance in terms of ST (71.62 and 77.42% for training and validation, respectively) in comparison with those presented by lighter birds (61.54 and 60.00% for training and validation, respectively). The capacity of the model to differentiate the 2 levels of the binary response was excellent (AUC ≥ 0.92) for both groups. On the other hand, the results in the training and validation sets for heavier broilers from SBY strain showed lower levels of MR (<9%) and FPR (<4%) compared with those for heavier HBY broilers. However, the ST levels for SBY strain (<58%) were lower than those for HBY birds (≥60%). The AUC values for heavier broilers from SBY strain (≥0.96) were comparable with those presented by lighter broilers from HBY strain. Note that the results for lighter broilers (<3.402 kg) from SBY strain were not shown due to the number of positives or carcasses with moderate or severe levels of WB condition was insufficient in this group to carry out the binary logistic regression analysis.

**Table 7:** Predictive performance of the selected binary logistic regression model for detecting woody breast (WB) condition in broiler carcasses by strain at different live weights using image measurements.

Strain	Live weight (kg)	Data set^1^	Fit details^2^							Parameter estimates and odds ratio (OR)				
			Gen. R^2^	RMSE	MAD	MR (%)	TPR (%)	FPR (%)	AUC	Parameter	Estimate	Std. Error	Pr > χ^2^	OR
High breast-yielding (HBY)	<3.402	Training	0.62	0.19	0.07	4.43	61.54	1.38	0.96	M1	2.27	1.03	0.0265	9.72
		Validation	0.74	0.16	0.06	4.76	60.00	1.72	0.99	M2	−9.37	3.38	0.0055	8.49 × 10^−5^
										M3	2.11	0.80	0.0086	8.26
	≥3.402	Training	0.62	0.31	0.19	12.41	71.62	6.25	0.93	M1	1.56	0.38	<0.0001	4.75
		Validation	0.61	0.32	0.19	11.50	77.42	7.32	0.92	M2	−8.92	1.52	<0.0001	1.34 × 10^−4^
										M3	1.75	0.35	<0.0001	5.74
Standard breast-yielding (SBY)	≥3.402	Training	0.57	0.24	0.11	8.09	56.25	3.33	0.96	M1	1.49	0.66	0.0243	4.43
		Validation	0.66	0.22	0.09	7.94	57.14	3.57	0.97	M2	−7.04	2.39	0.0032	8.77 × 10^−4^
										M3	1.54	0.53	0.0038	4.65

Abbreviations: AUC, area under the ROC (receiver operating characteristic) curve; FPR, false positive rate; MAD, mean absolute deviation; MR, misclassification rate; RMSE, root mean square error; TPR, true positive rate (sensitivity, ST).

1 Training (HBY, < 3.402 kg: n = 158; HBY, ≥ 3.402 kg: n = 266; SBY, ≥ 3.402 kg: n = 136) and validation (HBY, < 3.402 kg: n = 63; HBY, ≥ 3.402 kg: n = 113; SBY, ≥ 3.402 kg: n = 63).

2 The model used for WB prediction (model 1): Logit (p) = α + β1 M1 + β2 M2 + β3 M3.

Analyzing the odds ratio for each predictor variable (Table 7), the odds of observing an affected sample with WB condition were on average 4.59 times higher with 1-cm increase in M1, whereas the odds were on average 5.20 times higher with 1-cm increase in M3 of heavier broiler carcasses from HBY and SBY strains. On the contrary, the odds of detecting a carcass affected by WB abnormality became on average 99.95% smaller with 1-cm increase in the measurement M2 of heavier broiler carcasses of both strains.

The verification of the predictive performance of the selected model was evaluated by introducing the average for each measurement (M1, M2, and M3) in the prediction profiler. The results from this analysis based on broiler strain at different live weights are presented in Table 8. Regardless of the broiler strain and live weight, the actual WB status were consistent with their corresponding negative and positive predicted probabilities, which indicates that the model was effective predicting the WB condition in broiler carcasses. However, it is important to distinguish that the magnitude of predicted probabilities was variable. The negative predictive probabilities were high for all groups (≥0.96), whereas the positive probability was higher for heavier broilers from HBY strain (0.79) in comparison with lighter broilers from the same strain (0.69) and heavier broilers from SBY strain (0.53).

**Table 8:** Predicted probability^1^ of woody breast (WB) condition in broiler carcasses by strain at different live weights using image measurements.^2^.

Strain^4^	Live weight (kg)	Actual woody breast status^3^											
		No						Yes					
		M1 (cm)	M2 (cm)	M3 (cm)	Predicted probability (Yes)	Predicted probability (no)	Most likely	M1 (cm)	M2 (cm)	M3 (cm)	Predicted probability (Yes)	Predicted probability (No)	Most likely

HBY	<3.402	15.21	4.35	9.80	0.00	1.00	No	16.51	4.34	11.36	0.69	0.31	Yes
	≥3.402	17.73	5.05	11.37	0.04	0.96	No	18.74	5.00	12.83	0.79	0.21	Yes
SBY	≥3.402	17.44	5.19	11.02	0.01	0.99	No	18.80	5.21	12.87	0.53	0.47	Yes

1 The model used for WB prediction (Model 1): Logit (p) = α + β1 M1 + β2 M2 + β3 M3 by strain and live weight.

2 Means (No, HBY, < 3.402 kg: n = 203; No, HBY, ≥ 3.402 kg: n = 274; No, SBY, ≥ 3.402 kg: n = 176; Yes, HBY, < 3.402 kg: n = 18; Yes, HBY, ≥ 3.402 kg: n = 105; Yes, SBY, ≥ 3.402 kg: n = 23).

3 No = normal or mildly affected fillets by WB condition; Yes = fillets moderately or severely affected by WB condition.

4 HBY = high breast-yielding strain, SBY = standard breast-yielding strain.

In conclusion, the results from this study support the feasibility of the use of image analysis of broiler carcass features for detecting the WB condition, especially in big birds from high breast-yielding strains for the heavy debone market. Several subsets of conformational measurements (M1 through M4, and M6 as well as ratios M9 and M11) from broiler carcass images could be included in different statistical models to predict the WB myopathy; nevertheless, the best model included M1, M2, and M3, which suggests that conformation changes in broiler carcasses are mainly related to a breast width increase as WB severity increases. Even though the predictive performance of models for detecting WB condition using image measurements were associated with differences in WB incidence and severity rates among broiler strain, sex and age or live weight, it was found levels of MR lower than 19% with ST values greater than or equal to 50% and FPR levels less than or equal to 11% with good and excellent abilities to distinguish between the 2 levels of the binary response (AUC ≥ 0.89). Thus, it may be feasible to integrate these image measurements into commercial in-line, rapid and noncontact vision grading systems that would allow processors to identify broiler carcasses with WB to potentially sort, provide vast amounts of data downstream to further processing operations and upstream to live production in order to monitor factors associated with the development of WB myopathy.
